# Uses of Digital Mediation in the School-Families Relationship During the COVID-19 Pandemic

**DOI:** 10.3389/fpsyg.2021.687400

**Published:** 2021-06-25

**Authors:** Blanca León-Nabal, Cristina Zhang-Yu, José Luis Lalueza

**Affiliations:** ^1^Department of General, Developmental and Educational Psychology, Autonomous University of Barcelona, Bellaterra, Spain; ^2^Department of Psychology, Institute of Educational Research, University of Girona, Girona, Spain

**Keywords:** funds of knowledge, digital interaction, school-families interaction, schools on pandemics, mobile app uses

## Abstract

The COVID-19 pandemic has sharpened the inequalities in our societies. In Spain, we observed that the impact on schooling varied according to socioeconomic, gender and sociocultural variables. In this article, we present a case analysis illustrating the impact of the COVID-19 pandemic on schooling in early educational grades (ages 3–6), which leads us to focus on school-family relationship. First, we present some studies that show the inequalities in education during the lockdown period, the digital divide faced by both schools and families and how digital mediation impacts school-family relationships. Then we will introduce our study, which aims to explore the uses, potentials and limitations of an app intended to facilitate the relationship. Our study took place during September 2020-January 2021, when social restriction persisted. It took the form of a telematic ethnography in which we monitored the meetings of the Early Childhood Education teachers and their interaction with the families via an app-based communication tool. Results have allowed us to identify that most conversations are initiated by the school and their aim is to show families the classroom activities. We have also observed some advantages regarding the use of this app: communication can become more direct and immediate, and teachers have developed strategies to foster proximity in this relationship, as well as to respond inclusively to diversity. Regarding the challenges, we identified the lack of involvement of some families, the need to transform the roles played by families and children, and the difficulty to maintain personalized relationships.

## Pandemic and Digital Divide

The crisis generated by the COVID-19 pandemic has impacted schools around the world. There was a first phase (more or less prolonged, according to the circumstances in each country) characterized by home confinement and the cessation of face-to-face educational activities. This was followed by a second phase in which students returned to classrooms, but subject to restrictions and measures to ensure social distancing.

Focusing on the first phase, the physical closure of the schools in Spain took place without a contingency plan for the lengthy duration of this situation (in most European countries it persisted until the end of the school year, a period of 3–4 months). As a result, academic authorities and school boards had to improvise mechanisms for the continuing implementation of distance learning with the resources available at that time. The need to organize effective forms of distance learning severely tested schools' capabilities. On the one hand, the necessary technological resources for virtual two-way communication had to be assured, both in schools and in the students' homes (Bozkurt and Sharma, 2020). On the other hand, teaching methods had to be mediated by technologies appropriate for distance learning. In some cases, these methods had already been developed, but in others they had to be improvised (Schleicher, 2020).

The pandemic-induced school closures have aggravated social inequalities, as is starkly apparent in households' access to the resources that enable remote connection (Casado et al., 2018). According to the COTEC Foundation study, 66% of the students with a high socioeconomic level have three or more computers at home, compared to just 10% of those with a low socioeconomic level, most of which have only one (45%) or none (15%). Additionally, the gap in school digitisation is manifest from the ownership of the centers. While only 45% of the heads of public schools believe their teachers are trained to integrate digital devices into their classes, this figure rises to 69% in subsidized schools and 76% in private schools (Zubillaga and Gortazar, in press).

The data currently available suggest that the pandemic crisis is widening the gap in access to formal education, depending on the families' socioeconomic capacity and cultural capital and the schools' resources. In Catalonia (the region in which our research was conducted, at a school in Barcelona), Bonal and González (2021) revealed the impact of the pandemic on the educational and social divide. These authors conducted a macro-survey of over 30,000 families during the 1st months of lockdown. Their results show a digital divide that affects families of low socioeconomic status, which is manifest in indicators such as the availability of digital devices (in many cases there is less than one per person, or even just a single device, a mobile phone, for the entire family). This parameter is directly related to the time that students can dedicate to connected-at-home classwork. The statistic that best illustrates the impact on these families is that of “opportunities to learn,” reflected in variables such as support for home working, online communication with the teacher and the timely correction of homework by the teacher. The study found that 28.3% of the students in this socioeconomic group dedicated <1 h a day to school activities, lacked access to communication with the teaching staff and did not have their homework corrected. The authors also found that the degree to which families help their children with schoolwork, a factor that is especially relevant during lockdown as all class activity takes place in the home, and depends heavily on the parents' own educational background. Thus, 35% of the mothers who had only completed compulsory education helped their secondary school children, while 45% of the university-educated mothers assisted their children. The corresponding figures for the fathers were even more marked, at 16 and 32%, respectively. Family and extracurricular activities, which make a notable contribution to school skills, were also associated with the parents' social class.

These observations are derived from a study in which the population sample was biased by socioeconomic, gender and sociocultural variables. Since Bonal and González (2021) conducted the study during the lockdown, it was necessary to have internet connection, time and interest to respond to the questionnaire. This might explain why the participation of families with university-educated parents was significantly higher than that of the population. It would seem reasonable to hypothesize that the situation is much worse than the impression provided by this study, since it is precisely the families that suffer most from the digital divide that are least likely to have taken part.

During pandemics, school-family relationships became particularly important. Telematic classes challenged the physical space of learning (traditionally associated with schools) and homes became the new classroom. This means that families not only be able to afford internet connection and digital artifacts, but also appropriate space, equipment and environmental conditions for class participation. Parents became mediators of the teaching-learning process (the younger the child, the more important the role played by the parents), supervising communication with school and providing the material, intellectual and emotional resources necessary for the activity to take place (Trujillo-Sáez et al., 2020). In many cases, the scarcity of these resources was aggravated by the economic impact of the pandemic: parents had less time to support their children's school activity due to the need to seek alternative informal income, or because they remained in employment but performed essential low-skilled tasks, which were necessarily on-site.

Tarabini and Jacovkis (2020) conducted an online questionnaire during the lockdown. Answered by 2.777 teachers from Catalonia, the results in this report highlighted the social gap that affects schools, students and their families. According to their results, many families had access to the internet on their smartphones; while computers were much less frequently available (according to 34.5% of the teachers, very few of the families whose children attended their school had a computer in the house). Similarly, children were much less likely to have a digital device available for their exclusive use. This is consistent with the results on the participatory design research conducted by Gros et al. (2018) with migrant population in Barcelona: the participants are actually involved in our mobile-centric society (González-Patiño and Esteban-Guitart, 2014) showing that, despite of their socioeconomic situation, the smartphone is central in their lives. Moreover, the authors invite us to think not only in terms of access to technology and the internet, but the usage of it. This is the so-called second digital divide (Büchi et al., 2015). In this sense, the study by Gros et al. (2018) shows that the principal usage of the smartphone is social interaction and communication (by call or social networks) that connects them to their families and friends and facilitates daily life (such as e-mail or weather forecast).

### Innovation in School-Family Relationships and Digital Technologies as a Tool

In contrast to this gloomy picture, educational observers have reported that many schools, despite the difficulties encountered during the months of lockdown, have revealed great reserves of creativity, resilience, and innovation in coping with the situation (Cervantes Holguín and Gutiérrez Sandoval, 2020). This aspect of the real-world response is not reflected in the prior, quantitative-based research data that we cite, and which have proliferated during this period. In this line of argumentation, Iglesias et al. (2020) identify schools whose resilience to the crisis would be found in relation to the innovation processes that were underway before the pandemic.

In schools with most students from families that do not belong to the hegemonic culture or to wealthy socioeconomic backgrounds, these innovation processes often correspond to a rethinking of relationships with families. These new models start from a critical perspective of the deficient perspective of the low academic performing students and their families. According to the model of family-school discontinuities (Poveda, 2001), differences in performance can be explained by a lack of coherence between the values and interaction formats of school and family. This is because school practices and values are not neutral, but rather reproduce the hegemonic culture typical of well-off socioeconomic contexts (Tozer, 2000). To transform the school to be coherent with the family, it is essential to build fluid and close personal relationships (Poveda, 2001). Bronfenbrenner (1979), in his ecological theory, has highlighted the importance of collaboration, mutual understanding and agreement between microsystems -in this case, the school and the family- to favor human development -in this case of the boy or girl-, creating educational continuities between both.

However, family-school relationship is not built from neutrality, but within the framework of the school institution. Therefore, it is based on the parameters of the hegemonic and middle-class culture and, in addition, asymmetric power relationships come into play in these links (Whyte and Karabon, 2016), as well as the legitimation of a certain worldview and understanding of what school education is, and how families should be involved in it. Often, teachers identify family attitude as an obstacle to bonding with them (García-Bacete, 2003). Instead, teachers should assume the responsibility to build diverse and equitable relationships with families. This requires a progressive approach to co-responsibility, and an openness to transforming traditional school/family relationship formats (Collet-Sabé et al., 2014).

Consequently, it is necessary to promote accessibility so that families feel recognized and welcomed in school, so that they can utilize the knowledge currently invisible by the deficit paradigm (Esteban-Guitart and Vila, 2013a). In this sense, new models propose to leave behind a communication focused on negative aspects of the students, guaranteeing that there is also space to deal with their potentialities (Epstein, 2002). In addition, they require the creation of prior intersubjectivity between both agents that allows favoring two-way communication, information between contexts—that is, families can also share with the school their daily activities, tastes, values and knowledge (Oliva and Palacios, 1998; Epstein, 2002)—and mutual trust (Esteban-Guitart and Vila, 2013b).

In addition, moving toward the recognition and legitimation of marginalized cultures implies coping with the conflicts that may arise between school and family cultures (Tozer, 2000). It is necessary to accept that conflict is part of relationships, so strategies must be sought for its resolution with respect, dialogue and trust (Epstein, 2002).

One concrete proposal that aims to respond to the challenges presented by discontinuities between family and school is the funds of knowledge approach (González et al., 2005). From this perspective, it is assumed that all families (regardless of their origin, culture, language, religion, economic situation, etc.) have knowledge, skills and relationships. Therefore, they have resources that can contribute to academic learning. This approach is specified in the formation of a teaching team in ethnographic research -through a study group focused on funds of knowledge-, as well as in the implementation, by the teaching staff, of ethnographic visits to the homes of some families (Moll, 2014). These visits have the aim of identifying household knowledge so that they can be integrated into classroom life and generate curricular learning.

Various studies focused on funds of knowledge show how teachers become aware of their own prejudices toward families, of their position of power, of the ethnocentrism and racism that pervades the school institution and conceptions about upbringing (Zhang-Yu et al., 2020), etc. Therefore, through home visits, it is possible to question traditional and established teachers' roles as expert, modifying the asymmetry and enabling closer relationships (Whyte and Karabon, 2016).

However, given the current pandemic situation, approaches such as this are limited by the restrictions imposed. According to Iglesias et al. (2020), the situation caused by COVID-19 highlights social and educational inequalities that already existed. To respond to them, personal engagement and care are very important, as well as the need to diversify forms of relating to families. In addition, COVID-19 restrictions impose new forms of relationship in which digital technologies open up new possibilities. The great challenge is to identify, in this dystopian situation, the opportunities to accelerate changes toward educational improvement (Iglesias et al., 2020).

In recent years, the emergence of new digital technologies and their permanent and ubiquitous accessibility to information (Jenkins, 2006) has transformed our way of being, doing, learning and relating. Technology has become part of the day-to-day life of most school and family contexts. However, although technologies have entered the classroom, they do not usually transform teaching-learning processes and, much less, family-school relationships (Grant, 2009; Macià and Garreta, 2018).

There are many authors who defend the great potential that digital technologies can have to improve family-school relationships. Among the benefits that stand out, we highlight its potential for: (a) the creation of more open and collaborative educational communities; (b) the participation of those families that usually do not get involved in school (Vázquez et al., 2014); and (c) the creation of new contexts and patterns of knowledge, which favor the construction of more democratic and participatory schools (Aguilar and Leiva, 2012).

They can also contribute to the creation of a third space (Gutiérrez, 2008; Lalueza et al., 2019), that is, a hybrid space in which the different motivations of the participants (students, families and teachers) are legitimized, and in which, thanks to negotiation processes, shared learning objectives are created (Gutiérrez, 2008; Lalueza and Macías-Gómez-Estern, 2020). Finally, this approach increases the quality of communication so that all members of the school community become producers, not merely consumers or receivers of knowledge (Fischer and Bloomfield, 2019).

However, desirable there is also agreement that these potentialities are not used in practice (Beneyto-Seoane and Collet-Sabé, 2016). Some of the obstacles identified to using digital technologies in a transformative way in family-school relationships are the lack of infrastructure or material, inadequacy of training (both for families and teachers), inequalities of access, and unfavorable attitudes toward these resources (Beneyto-Seoane and Collet-Sabé, 2016; Macià and Garreta, 2018). Finally, the lack of reflection and evaluation on the use of these tools also limits their transformative potential (Grant, 2009).

However, given the current pandemic situation, what seemed like a desirable option has become an imperative to promote continuity of family-school relations. Digital technologies are currently an essential channel for communicating and connecting with families. Therefore, it seems relevant to highlight some fundamental ideas on how to use these tools in a transformative way.

## Context of the Research

This study has been developed in a public school located in the Bon Pastor neighborhood, a working-class environment in Barcelona, Spain. Most of the students (aged 3–16 years old) belong to Roma ethnicity, or are descendants of African, Asian or Latin American migrants.

This study has been conducted in the framework of a longer-term action-research programme (Lamas and Lalueza, 2012; Lalueza et al., 2020; Lamas et al., 2020; Walker et al., 2021). For almost 2 years before the declaration of the pandemic we had been implementing a Funds of Knowledge exercise as a strategy to transform school-family relations within Early Childhood Education. Due to this experience and our positioning within the school, we were able to follow events *in situ* during the lockdown and after. So, the researchers had direct access to the school activity during the lockdown, monitoring the teachers' meetings regarding the strategies to be adopted by the school for virtual relationships with the children's families.

When the state of alarm was decreed in March, schools were suddenly faced with the need to connect the teachers, the children and the families, in their respective homes. At the school examined in this study, the teachers were contacted and coordinated by e-mail and telephone, but these channels were soon replaced by videoconferencing. Connection with the families was first established by telephone. The management team called each family, one by one (only one family could not be located by this means), and this initial contact served to identify the children's main needs.

The first necessity was to respond to basic nutritional demands, since many of the children had been receiving free school meals, to ensure a nourishing, balanced diet. This service had been compromised by the closure of the school. Furthermore, many families were in a precarious economic situation, having been deprived of their livelihood by the lockdown. The distribution of food vouchers was a major priority during the first round of contacts, as the school considered the children's welfare to be its primary function, well-ahead of academic concerns.

Secondly, the school addressed the emotional and social needs arising from the enforced isolation. Telephone conversations informed the school about the families' situations, how they were coping and about the lack of space and resources that many were experiencing. Paradoxically, the absence of face-to-face contact enabled us to learn more about the lives of these children and their families. Many families expressed their appreciation of the school's efforts: just making contact and expressing the school's concern was warmly welcomed and, the school managers believe, reinforced mutual trust.

The third priority was to draw up guidelines for action and challenges that would keep the students focused on goal-oriented activities. Accordingly, strict adherence to the school curriculum was the last of our concerns. Thus, according to Tarabini and Jacovkis (2020), while at many of the schools attended by children from middle and upper-class families, the school's connection with families at home was aimed at maintaining curricular activity, in our case, issues such as food availability and emotional well-being took center stage, relegating curricular learning to a secondary consideration.

As commented above, the first medium used to relate with families was that of the telephone. This channel was necessary and irreplaceable for making the first contact, but is inefficient for long-term communication, since it requires a synchronous connection for each family. Hence, new forms of interconnection emerged, based on the use of mobile phones. Very few of the families had access to a PC, laptop or tablet, but all had at least one mobile phone, and this instrument provided a resource and a dimension that was both “natural” and radically different from landline-based phone calls.

Furthermore, the use of mobile phones as a means of communication was supported by pre-lockdown experience, so that we were aware which approaches and methods worked and which did not. In the analog experience, course meetings, parent-teacher association meetings, interviews unrelated to specific problems and printed circulars were all ineffective as channels of communication. Similarly, among digital resources, neither the school's website nor its use of e-mails proved satisfactory. On the other hand, just as in face-to-face activity before the pandemic, meetings at the school gates at the beginning or end of the school day, activities related to the presentation of the students' audiovisual output and meetings arising from the Funds of Knowledge programme all worked well. Also effective, in digital communication, were Instagram and a communication app called Dinantia^1^

Prior to the pandemic, Instagram had served as a showcase for activities carried out within the school and during outings. During the lockdown, it provided a tutorial for families with children in Early Childhood Education and in the 1st years of primary school. It also constituted a hub of proposals for family activities. The mobile app, on the other hand, was a one-way communication tool that had previously been used as a substitute for the school's provision of printed information to families. During lockdown, it was activated as a bi-directional tool, enabling a permanent means of communication via messages, files, images and videos.

## Method

### Aim

The aim of this study was to identify the uses of digital communication channels in school-family relationships, the opportunities offered and the limitations that may be encountered, in the Early Childhood Education grades of a public school in a low-income environment.

### Data Collection

Our study took place during the period September 2020-January 2021, and took the form of a telematic ethnography in which we monitored the meetings of the Early Childhood Education teachers and their interaction with the families via an app-based communication tool.

The research team held three virtual meetings with the teachers (in September and October 2020 and in February 2021), and one researcher attended the twice-weekly meetings of the Early Childhood Education teaching team during November, December and January. In every case, a field diary was used to systematically collect information about the school's relationship with the families. In addition, we had access to the smartphone app, which allowed us to observe and analyze the communication established between the families of one of the classes, the class tutor (who was also the grade coordinator) and the school. This class was made up of 17 children, corresponding to 15 families (there were two pairs of siblings). Specifically, we had access to all the group messages (addressed to all the families in the community or in the class), throughout the term, individual messages (i.e., those exchanged between a single family and the teacher) and institutional messages (generated by the management team) sent in November and December 2020. In addition, we held two virtual meetings with the grade coordinator which helped us understand the school operation. The content of these meetings was included in the field diary, as well. Finally, we conducted a face-to-face semi-structured interview with the same coordinator, discussing the limitations and potential of the resources for managing relationships with families in the current situation. This interview was recorded on audio and transcribed for later analysis.

### Analysis

The information obtained from interviews, the monitoring of teachers' meetings and the messages sent via the app were transcribed and analyzed as textual data. For this purpose, data were categorized into three thematic blocks, all interrelated but focusing on specific areas of interest. For each of these blocks, the research team formulated a series of questions guided by the theoretical framework (see Table 1).

**Table 1:** Data categorization.

**Thematic blocks and theoretical questions**	**Dimensions**
**Block 1: School-family continuity in a context of cultural diversity and social vulnerability**	
**Do current practices related to the school-family relationship and to the use of digital technologies allow or favor…**	
•… awareness of the deficit paradigm and the adoption of a critical attitude in this respect? •… awareness of the ethnocentrism and racism of the school institution (and of us all, individually), spurring us to transform these outlooks? •… awareness of the power relations present in the family-school relationship, especially in contexts of discontinuity, and their transformation into reciprocity?	Focusing on the families
•… the existence of two-way communication and information exchange between the contexts of school and home? •… the identification, appreciation and legitimization of sociocultural practices, values, languages and relationships that differ from those of the hegemonic culture? •… the socio-interactive repertoires of all cultural groups involved to be incorporated into classroom life?	Bidirectionality
•… the evaluation and improvement of all these practices?	Evaluation
**Block 2: School-family continuity via relational and affective factors**	
**Do current practices related to the school-family relationship and to the use of digital technologies allow or favor…**	
•… the existence of smooth, continuous communication?	Access to teachers
•… families' feeling comfortable, welcomed and recognized in their relations with the school? •… mutual trust? •… interaction not only regarding conflictive questions, but also positive ones? •… the recognition and resolution of conflicts through dialogue?	Affectivity
•… contextualizing school-family relations according to the characteristics of each family? •… the school to improve its relationship with all families? •… the construction of diverse and equitable relationships, offering families diverse ways, times and strategies for communicating with the school and enhancing their involvement? •… the inclusion of every family, not just those which are easily addressed?	Attention to diversity
•… the evaluation and improvement of all these practices?	Evaluation
**Block 3: School-family continuity via digital technologies**	
**Do current practices related to the school-family relationship and to the use of digital technologies allow or favor…**	
•… building a more participatory, open and democratic educational community? •… families to become producers of content and not just recipients? •… families to become involved in decision-making regarding the use of mobile app?	Participation by the families
•… learning contexts to be connected via the creation of a third space? •… children's playing an active role in family-school mediation?	Connection between learning contexts
•… greater efficiency in communication?	Efficiency
•… the evaluation of mobile app as a means of enhancing the family-school relationship?	Evaluation

The researcher who conducted the fieldwork (León-Nabal) identified each textual quotation categorizing it as a response to one of the questions in the table, and this assignment was later reviewed by the other two team members. After discussion, the final category system was agreed upon by consensus. So, the category system did not arise from three independent coding in origin, a limitation according to Anguera et al. (2018), but we have prioritized the continuity between fieldwork and analysis, privileging the researcher's gaze in her interaction with the investigated object.

Since not all the citations correspond to the use of the mobile application, the object of the study presented here, those that did refer to this tool were selected, and together they were grouped for presentation into three groups: (a) uses and contents of communication, (b) strategies used and perceived advantages, and (c) difficulties and challenges encountered.

## Results

### Uses and Contents of Communication

In the present school year, family-school relationships for children in Early Childhood Education (3–6 years) have been limited almost entirely to contacts via the smartphone app. Beyond this instrument, teachers and families have only met at the school gates, at the beginning or end of the school day (briefly and with social distancing) and in personal interviews (only face-to-face when strictly necessary). Other resources such as telephone calls or e-mail have been used only occasionally.

Although this app was first used in 2019, the possibility of bidirectional messages (so that families could also send messages and interact with their children's teachers) was not activated until lockdown began. In the school year 2020–2021, this facility was maintained, although the platform does not allow families to interact with each other. The mobile app is used for different types of messages, depending on the sender and to whom it is addressed:

- General messages: Notifications for the entire educational community (such as the school meal menu for the coming month or news of COVID cases in the school).
- Group messages: Addressed to all the families and teachers of a school year, age group or class. At least one is sent every Friday to report—through a text, images and/or video—the week's activities in class. The school also sends out a “news bulletin” advising families of upcoming important events.
- Individual messages between a family and the teachers, for example, to notify of inability to attend. In addition, every month or two, the teachers send the parents individual or small group photos of their children.

The frequency of each use is detailed in Table 2):

- in-school activity (showing families the activities carried out in the classroom and elsewhere in the school, as well as messages sharing the school's weekly music schedule).
- at-home activity (showing the teacher an activity carried out in the family context).
- gratitude (expressing thanks, for work done, photos sent or any other detail appreciated).
- mood (describing the child's mood or attitude in a certain situation, for example, if they were crying on being left at the school gates, reporting that they have since calmed down).
- logistics (communicating information related to the timetable and/or calendar, materials that need to be brought to school, personal image rights, confirming an appointment for a personal interview or reporting the child's absence from school).
- school meals (informing families of the school's menu for the next month).
- health (reporting symptoms, wounds, the presence of lice or a positive case of COVID in the school).
- theory (sharing information related to developmental and educational theories).

**Table 2:** Content of mobile app messages according to sender/receiver status.

**Type**	**Frequency**	**Content**	**Frequency**
General	10	School activity	3
		Logistics	3
		Health	2
		Monthly menu plan	2
Group	25	School activity	19
		Logistics	4
		Health	1
		Theory	1
Individual initiated by the school	9	State of mind	3
		Logistics	3
		Health	2
		Thanks	1
Individual initiated by the family	21	Logistics	11
		Thanks	5
		Health	2
		Home activity	2
		State of mind	1

With the app, it is usually the school that initiates conversations with the families (at the general, group or individual level). The main aim of group messages is to show families what their children are doing at school, using text, photos and videos. However, only six of the 15 families in the group have read all these weekly messages, while another five have done so practically every time (leaving no more than three messages unread). On the other hand, one family did not read any messages until November and another started in December. There were two more families who did not read any message at all. However, one of them was not taking his child to school. This family has recently informed the school that he was transferred to another center.

Regarding messages initiated by the families, 8 out of the 15 families in the class sent messages to the teacher during November and December. In total, the families initiated 21 individual conversations with the teacher, although five of these were in response to a group message with photographs. Most of these messages (11 out of the 21) concerned logistical issues such as dates, timetabling or absences. In addition, there were five messages of gratitude for the images sent by the teacher, two referring to health issues, two showing the teacher the child's activities in the family context and one regarding the child's state of mind when entering school.

### Strategies Used and Perceived Advantages

Our analysis of the contents and use of app mobile and of the regular meetings between teachers, together with the interview with the research coordinator, highlights the advantages of this type of communication, and reveals the strategies developed and used by the teaching team in their relation with the students' families, in response to the needs and challenges that have arisen in the present circumstances.

In the first place, according to the grade coordinator, families enjoy greater access to the teachers, who can obtain direct, immediate communication with their child's tutor, without intermediaries, albeit restricted to written and virtual formats (and although other, face-to-face, channels have been lost).

“*Families know they can write to the teacher whenever they want. It's me who reads their messages, there are no intermediaries, it's more direct”* [Coordinator_Interview_16.12.20].

Secondly, despite the inevitable limitations imposed by the virtual format on the “personal touch,” some strategies can be used to foster proximity in the relationship.

For example, sometimes children are agitated or crying when they arrive at school; on three occasions when this happened, the teacher sent the parents a message and a photo which showed the child playing after calming down. For instance:

“*Good morning*. ^***^ *is fine now and she is playing happily, as you can see in this photo. We'll see you at 12.30*.”

Attached file: Photo of the child in the classroom.

[App_IndividualMessage _11.11.20].

Emoticons can also accompany the text. For example:

“It's nothing serious, but he'll come out today with a few scratches”

[App_IndividualMessage _30.11.20]

Communications via the app always show the students and their classroom activities in a positive light. According to the school's head teacher, in an interview conducted in July 2020, previously it had been difficult to find the right moment to let the families know about the things that were going well. So, the mobile app may have generated new communication opportunities with the families about the positive aspects of their children's schooling.

Thirdly, teachers have developed strategies responsive to the diversity of these families. When the educational focus is on inclusion, the school must offer different responses according to the needs, potential, interests and characteristics of the children and their families.

Within the app itself, the teacher uses strategies to contextualize and personalize her messages, thus addressing the diversity of families. One of these strategies consisted of translating the messages into Spanish, since the child's mother did not understand Catalan. Another strategy was to replace the weekly group message with an individual message with photos -in which the child appeared either alone or in a small group- to each family. This kind of message was sent twice and was answered by five families with a message of thanks. Prior to this innovation, none of the group messages had received replies. Virtually all the group messages included not only text but also emoticons, illustrations and images, and sometimes videos, thus diversifying the ways in which information was provided.

“*Our families really like to see photos, which is why in all our weekly group messages we send them photos or videos.“* [Coordinator_Interview_16.12.20].

However, the teachers have had to face another challenge: some families do not use the app (they do not read or do not reply to the messages), despite its being one of the most important channels of communication between the school and the families. The teachers have overcome this problem with two of the four families involved by taking advantage of the few moments of face-to-face contact (during interviews and at the school gates during the children's entry and exit) to demonstrate the app and encourage its use.

“*For example, if a form needs to be signed, the parents do it using Dinantia. But we let the families know about this in person, when the children are coming out of school, and explain how to do it.”* [Coordinator_Interview _16.12.20].

### Difficulties and Challenges Encountered

The current situation, which is both uncertain and complex, is posing new challenges to the development of a good understanding between the school and the families, and to ensuring continuity between the two main educational contexts—learning and activities—in Early Childhood Education. Although this school has successfully developed new ways of communicating with the children's families, there remain areas where further reflection and improvement are needed. Challenges we have found involve (a) the level of participation and quality of communication, (b) transformation of roles played by families and children, and (c) maintenance of personalized relationships.

The first of these major challenges mainly concerns the families who do not read the messages sent. As we have shown, two of the 15 families did not read any of these messages. According to the teacher in question, various explanations might account for their non-involvement. On the one hand, these families may lack access to infrastructure or material resources:

“*Perhaps the family's mobile phone has broken, or they haven't got one, or now they do, now they don't, or the number has changed”* [Coordinator_Interview_16.12.20].

Alternatively, according to the teacher, there may be a lack of interest in school matters:

“*I think the reason why this family doesn't read the messages is just that they can't be bothered, it's because they think: “If it's just so you can tell me to bring an apron, well, I'm not going to bring it anyway.” That's the attitude of this particular family. (…) Maybe they would like to have the photos, but they don't want to do all that's necessary: put your contact details on their phone, download the app, all the rest. On the other hand, they do know how to do tiktoks. I think it's just they're not very interested in school affairs.”* [Coordinator_Interview_16.12.20].

Even so, perhaps we should reflect on whether this lack of involvement with the school might have arisen from the wide gap that still exists between the school's and the family's forms of socialization, as has been suggested by Collet-Sabé et al. (2014).

Finally, the scant involvement of some families in reading the messages and in interacting via the mobile app might be due to a simple lack of digital competence:

“*The coordinator told me that some families lack digital skills, and so it's difficult for them to use the app correctly, even after it was explained to them and after she went over to show them.”* [Blanca_FieldNotes_27.11.20].

A second challenge is to transform and re-assess the roles played by families and children in this relationship. As we have seen, although some families use the app to interact with the teachers, this technological resource is not generally used by families to share their activities, preferences or family understanding with the school. Moreover, in the only two cases in which families have shown the school something about their household, this was, according to the teacher:

“*It's only because I insisted, at the school gates. They told me about it (for example, the child's handicraft work) and I said, Why don't you send me a photo? I do try to create some kind of link, a relationship, but this doesn't work, either.“* [Coordinator_Interview _16.12.20].

The same teacher, when asked why the parents didn't often send these types of messages, observed:

“*Maybe it's because they don't know? Perhaps they think, What can I send the teacher? Maybe they're just cautious and don't want to bother us.”* [Coordinator_Interview _16.12.20].

On the other hand, the way teachers use language through the app also influences the role played by the family in this interaction, since it may accentuate the asymmetric power relations between the family and the school. In this sense, we have identified situations in which the teacher addresses the family using a formal, technical register, one that is more typical of the hegemonic school culture, and which might be difficult for many families to understand:

“*The preventive use of insecticide products is not recommended for persons not affected by pediculosis. (…) They are sometimes confused with dandruff and seborrheic scales, but neither of these formations is attached to the hair and they are easily removed. Moreover, dandruff does not produce the pearly shine of nits.”* [App_GroupMessage_9.11.20].

”*The constructions are not only for play, they also teach us a multitude of mathematical and physical concepts, such as geometric shapes, series, sizes, comparisons and classifications“* [App_GroupMessage_20.11.20].

We have also identified messages in which proposals were addressed to families about how they should interact with their children, from a clearly ethnocentric perspective:

“*Now that Christmas is almost here, and we're all thinking about what toys to get our children, we want to help you consider how to enrich their games, with guidelines that will encourage them to grow, learn and develop without falling into the traps of sexist and gender-role stereotypes, and thus promote equality.”* [App_GroupMessage_22.12.20].

Such communications were sent to the whole group, in the form of information bulletins, and contrast with the warmth of the personal messages intended to keep the families in touch with each other. These “cold” uses of language perpetuate asymmetric power relationships, thus generating further inequality.

These considerations lead us to reflect on the roles played by families in determining the use made of the mobile app. In this case, the families were not part of the process; it was school personnel who decided to introduce the app, and decided how it should be used—initially to replace printed handouts, and currently as a two-way communicative tool enabling teachers to show the families what their children do at school.

A third challenge presented by the pandemic situation consists of constructing emotional ties with families. Although the teachers do their best to maintain close relationships through the virtual format (as described in the previous section), it can be difficult to maintain affectivity in the relationship when there are no face-to-face encounters:

“*This year is different, there's less bonding, before there was more. We've lost this good relationship.”* [Coordinator_Interview_16.12.20].

“*Written messages are very impersonal; a message could be interpreted in many ways. Moreover, we have families who don't read the messages or who do read them but don't usually reply.”* [Coordinator_Interview_16.12.20].

Finally, to make changes in the relationship with families, it is necessary to have moments and spaces for evaluation of and reflection on one's own practice. The practice of the Knowledge Funds project involved dedicating two sessions a month to reflect on relationships with families, which was interrupted by the pandemic situation.

“*This year we haven't got anywhere we can consider and evaluate our relationships with the families. The Funds of Knowledge meeting was the only time. Yes, we have the evaluation meetings, but then we talk more about the children than about their families. Then, if specific problems arise, the matter is presented to Coordination, but these tend to be one-off incidents."* [Coordinator_Interview_16.12.20].

In their weekly meetings the teaching staff do talk about relationships with families and the use of the app. However, these conversations are spontaneous and refer more to the content of the weekly messages, and do not constitute an intentional, planned reflection on their own practices, or their beliefs and prejudices about the families, the use of digital technology in this relationship, school ethnocentrism or power relations.

## Discussion

Our study has afforded us a glimpse at 5 months of use of a technological platform in a school belonging to a low-income social environment, with students belonging to minoritized groups, in a context of restrictions produced by the pandemic. The results allow us to reflect on the potential and limitations of this type of tool in the development of relationships between school and families.

First, the results indicate that there is a need for convergence between the use of these devices and face-to-face encounters. During lockdown, the children's needs for attention and affection were the most important factor, and digital support (especially for families whose situation is precarious) is still inadequate as a means of mediating relationships with a strong emotional component. This deficiency has led to technologies necessarily being complemented by opportunities for face-to-face meeting, which continues to be the priority for affective communication. Our results highlight the reality of a convergence between face-to-face meetings (even if only fleeting exchanges at the school gates) and the use of the mobile app. This mixed construction of school-family interactions seems to follow the same trend as that of teacher-student educational interactions, in line with the hybrid education model (Pardo and Cobo, 2020), who call for the traditional architectural barriers between presential and virtual education to be overcome. Digital devices offer great potential for the expression of feelings, which could undoubtedly be developed in tools like the app mobile to expand the range of interactions provided. Nevertheless, it seems that the ultimate meaning of these interactions is still constructed via physical encounters, suggesting we should continue to reinforce the complementarity between digital uses and face-to-face encounters in the construction of relationships, and not neglect their emotional basis.

The most interesting finding in relation to the potential of the new tools is their *multimodality*. The use of images and videos in the app produces a qualitative change in the messages exchanged between the school and the families, by introducing “fragments of life.” Prior to the pandemic, family-school relationships were mediated by the normative use of printed communications and the punitive one of appointments to resolve conflicts or to discuss shortcomings. With the novel situation and the use of the mobile app, this relationship has been transformed into a dialogue on everyday experience, with the child as an active protagonist. Interestingly, this medium reveals the inadequacy of certain formal uses of language that are alien to the families' habitual forms of expression. Multimodality, on the other hand, seems to be more attuned to everyday discourse, in which words are contextualized by images and activities. The main limitation observed in this respect is that the app is used almost exclusively at the initiative of the school, as the initiator or explicit requestor of the exchange.

Another important potential of digital platforms is the possibility of increasing the agency of families through the creation of shared content. New possibilities have arisen, but to date there has been very little development in this area. A virtual space enabling content creation by families would promote the legitimation of their knowledge, as is intended with the Funds of Knowledge approach (González and Moll, 2002; Hogg, 2011; Esteban-Guitart et al., 2019), but, as discussed above, agency continues to reside mainly with the school, which still generates most digital content and defines the areas in which families may contribute. Digital devices, in theory, offer bidirectionality, but in practice this potential is largely unseen. In short, if there are few opportunities for families to share their socio-cultural practices, values, forms of relationship or socio-interactive repertoires with the school, this makes it difficult for the school to identify, value and legitimize these factors, incorporating them into classroom life and fostering continuity between the two contexts. Therefore, from the outset the families have been mere recipients, and not producers or agents of this change, and have had to accept the decisions made beforehand by the school.

The same “recipient” role is apparent in the use and content of the messages: the families receive the information that the teachers send, but in most cases they do not use the app to produce new content and share it with the school. Very probably, as the coordinator suggests, this is because they are overly prudent and do not believe it appropriate to share their own knowledge and practices. In addition, the fact that families can only address the teacher (and not each other) limits the possible uses of this technology. The logic of mobile apps aimed at constructing social networks should allow these families to conduct group interactions, sharing their knowledge, ideas and activities, thus helping create a more open, democratic and participatory community. On the other hand, this is a complex process that poses dilemmas and risks and might generate some apprehension among the teachers.

Furthermore, it would be useful to analyze the role played by the children in mediating between the school and family contexts via technology, since they are the real protagonists in this relationship. From the teachers' observations, and our analysis of the messages sent via the app, it seems clear that the children are being left on the sidelines in this respect. Only when the parents read the messages together as a family are the children able to mediate in the process, by explaining the photographs showing the classroom activities.

To overcome these limitations, we have the great potential of mobile apps fostering *participation* and the configuration of communities of interest (Lacasa, 2020) and affinity spaces (Gee, 2013). In a context such as that of the school examined in this study, a suitable app could help generalize participation and enhance the development of community networks with digital support. On the other hand, this process might be obstructed by the formal dynamics of the school institution, weighed down by its established, legitimized unidirectionality as a repository of normative knowledge. Moreover, the configuration of the digital device as a mechanism that places the school at the center of attention, connecting it with the families on the periphery and excluding any kind of *horizontal* interaction, impedes the creation of the kind of community found in other social networks.

Another potential of the telematic devices is the *distribution of knowledge*. This attribute meshes perfectly with one of the goals of the Funds of Knowledge approach, namely to foster the construction of a meeting space between the school's knowledge and that of the families. The mobile app provides an opportunity to digitally re-create this space, to nourish it with contributions from the school, to provide a showcase for the children's activities, to lend them meaning and to include contributions from the families, thus legitimizing the domestic practices that guide the children's development. The strategies discussed above reveal potential avenues to explore in this direction, but also highlight the great distance remaining to be covered and the limitations of the digital apps currently available. In our opinion, three important lines need to be developed.

Firstly, in line with the Funds of Knowledge study group (Esteban-Guitart et al., 2018), teachers could collectively and critically evaluate the use of the tool within the framework of strategies to develop new forms of school-family relationships. In addition, they should consider issues such as the attitudes taken toward families, ethnocentrism, power relations, channels of communication and the uses of technology in this respect. Indeed, this process is already under way, within the framework of the Funds of Knowledge approach (Zhang-Yu et al., in press). The mobile app, as a mediating device, transforms activities, but the conscious definition of the goals motivating these activities is what actually regulates the directions taken in this transformation.

Secondly, as we have seen, this digital-driven communication must become truly bi-directional, to transform it from a medium by which information flows primarily from the school to the families to one in which both directions are active. Meeting this challenge, however, will require imagination and dialogue. Furthermore, the very architecture of the tool can facilitate or limit the flows. Therefore, we need to consider the possibility (which has already been accomplished, technologically, in social networks) of achieving horizontal communication between families, and thus exploiting the potential offered for concerted, collaborative action. This advance would also give families a voice in how technology is used in their relationships with the school.

Finally, students must become actively involved in family-school mediation. Their appropriation of digital technologies would accompany that of the motives and goals of school practice, granting them agency in promoting educational continuity between these two contexts.

However, some study limitations should be noted when considering these conclusions. Since it is a case analysis, results describe a contextualized and concrete reality and cannot be generalized. Besides, data categorization is not objectively replicable. Thus, the above proposals should only be considered the starting point for future action taking into account the experience described, our analysis of the situation and the suggestions made for improvement, from our discussions with the teachers involved. This account, therefore, represents the conclusion of one stage of the process and, at the same time, an introduction to new spaces for reflection, new challenges to be addressed and lessons to be learned. With this study, we hope to have contributed to a greater understanding of the school-family relationship during a period in which social inequalities have been exacerbated. The pandemic has spurred us to re-examine the ways in which schools and families interrelate and communicate, but further research and fresh thinking are undoubtedly necessary to meet the present and future challenges to our society.

## Data Availability Statement

The original contributions presented in the study are included in the article/supplementary material, further inquiries can be directed to the corresponding author/s.

## Ethics Statement

The studies involving human participants were reviewed and approved by Research Ethics and Biosafety Commitee (CEBRUdG) Universitat de Girona. The patients/participants provided their written informed consent to participate in this study.

## Author Contributions

All authors listed have made a substantial, direct and intellectual contribution to the work, and approved it for publication.

## Conflict of Interest

The authors declare that the research was conducted in the absence of any commercial or financial relationships that could be construed as a potential conflict of interest. The reviewer VV-H declared a shared affiliation with one of the authors, BL-N, to the handling editor at time of review.

## Footnotes

^1^https://www.dinantia.com/en/.
